# Gestation age-associated dynamics of mitochondrial calcium uniporter subunits expression in feto-maternal complex at term and preterm delivery

**DOI:** 10.1038/s41598-019-41996-3

**Published:** 2019-04-02

**Authors:** Polina A. Vishnyakova, Nadezhda V. Tarasova, Maria A. Volodina, Daria V. Tsvirkun, Iuliia A. Sukhanova, Tatiana A. Kurchakova, Nataliya E. Kan, Marzanat K. Medzidova, Gennadiy T. Sukhikh, Mikhail Yu. Vysokikh

**Affiliations:** 1 National Medical Research Center for Obstetrics, Gynecology and Perinatology named after Academician V.I. Kulakov of Ministry of Healthcare of Russian Federation, 4, Oparina st., Moscow, 117997 Russia; 2 Molecular Medicine Institute, I.M. Sechenov First Moscow State Medical University, Moscow, Russian Federation, 8, Trubetskaya st., Moscow, 119991 Russia; 3 National Research University Higher School of Economics, 20, Myasnitskaya st, Moscow, 101000 Russia; 4 Lomonosov Moscow State University, Biology Faculty, 1/12, Leninskye gory, Moscow, 119234 Russia; 5 Belozerskii Institute of Physico-chemical Biology, Lomonosov Moscow State University, 1/40, Leninskye gory, Moscow, 119234 Russia

## Abstract

Calcium plays a role of universal cellular regulator in the living cell and one of the crucial regulators of proper fetal development during gestation. Mitochondria are important for intracellular calcium handling and signaling. Mitochondrial calcium uniporter (mtCU) is a multiprotein complex of the mitochondrial inner membrane responsible for the transport of calcium to the mitochondrial matrix. In the present study, we analyzed the expression level of mtCU components in two parts of the feto-maternal system – placenta and myometrium at full-term delivery and at preterm birth (PTB) on different stages: 22–27, 28–32, 33–36 weeks of gestation (n = 50). A gradual increase of mRNA expression and changes in protein content of MCU and MICU1 subunits were revealed in the placenta during gestation. We also observed slower depolarization rate of isolated placental mitochondria induced by Ca2+ titration at PTB. In myometrium at PTB relative gene expression level of MCU, MCUb and SMDT1 increased as compared to full-term pregnancy, but the tendency to gradual increase of MCU protein simultaneous with MCUb increase and MICU1 decline was shown in gestational dynamics. Changes observed in the present study might be considered both natural dynamics as well as possible pathological mechanisms underlying preterm birth.

## Introduction

**Figure 1:**
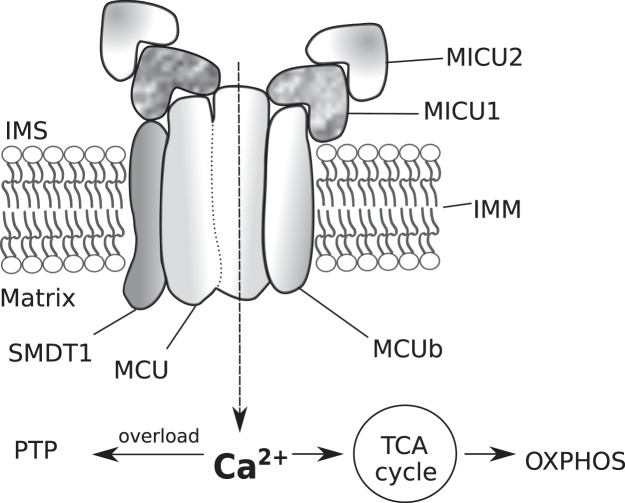
Schematic representation of mitochondrial calcium uniporter complex components and effects of Ca^2+^ import. IMS – intermembrane space; IMM – inner mitochondrial membrane; MCU – mitochondrial calcium uniporter protein; MCUb – mitochondrial calcium uniporter regulatory subunit b; MICU1, MICU2 – mitochondrial calcium uptake protein 1 and 2; SMDT1 – single-pass membrane protein with aspartate-rich tail 1 (or EMRE - essential MCU regulator); TCA cycle – tricarboxylic acid cycle; OXPHOS – oxidative phosphorylation; PTP opening – permeability transition pore opening.

Сalcium ion (Ca^2+^) is a key player in extracellular signals transduction and regulation of activity of target cells. Different calcium-binding proteins, which are located in cellular organelles and cytoplasm, decode the signal from local increase/decrease of calcium concentration and trigger an appropriate molecular cascade^1,2^. Despite the leading role of endoplasmic reticulum in storage and release of calcium, mitochondria are also involved in the maintenance of calcium homeostasis^3–5^. Calcium ions are necessary for proper functioning of aerobic metabolism: pyruvate dehydrogenase^6^, three Krebs cycle dehydrogenase are activated by Ca^2+^ ^7^. Besides of its influence on cellular bioenergetics, mitochondrial calcium also plays an important role in cell death regulatory system through the calcium-induced permeability transition pore opening and apoptosis induction^8^. Calcium ions are imported into cell through the plasma membrane channels or ion exchangers and stored in endoplasmic reticulum and mitochondria^9^. Calcium import through the outer mitochondrial membrane occurs by means of the voltage-dependent anion channel (VDAC)^10,11^. Mitochondrial calcium uniporter (mtCU), located in the inner mitochondrial membrane, transports calcium from intermembrane space to the mitochondrial matrix^12^. MtCU is a protein complex (Fig. 1) consisting of pore-forming subunit – MCU and several regulatory subunits (MICU1, MICU2, MCUb, SMDT1 (or EMRE))^13–15^. Mitochondrial calcium uptake 1 and 2 (MICU1 and MICU2) subunits were identified as crucial MCU regulators which determine calcium concentration threshold and cooperative activation of MCU^16,17^. In particular MICU1 plays a role of gatekeeper in MCU-mediated calcium uptake and protects mitochondria from Ca^2+^ overload^18^. Single-pass membrane protein with aspartate rich tail 1 (SMDT1) was shown to be essential for stabilization of MCU–MICU1 interaction and for prevention Ca^2+^ ions leakage through MCU channel at the baseline state of the cell^19,20^. MCUb acts as dominant negative MCU regulator, reducing calcium level elevations in mitochondrial matrix after mtCU stimulation with agonists^19^.

Calcium plays an important role both during pregnancy and in labor induction^21^. It serves as a structural component in form of hydroxyapatite during bone formation and as a secondary messenger molecule that induces different signaling pathways, which lead, for example, to uterus contractions^22^.

A number of studies revealed the link between abnormalities in calcium homeostasis and such gestational pathology as preterm birth (PTB)^23,24^. PTB is defined as birth occurring before 37 weeks of gestation and it remains a leading cause of perinatal and neonatal morbidity and mortality^25^. Every year about 15 millions of babies are born preterm^26^. At last, PTB is accompanied with further complications for both mother and fetus^27^. Basing on gestational age PTB is divided into three sub-categories: extremely preterm (<28 weeks), very preterm (from 28 to <32 weeks) and moderate to late preterm (from 32 to <37 weeks)^28^. Main causes of PTB are increase of pro-inflammatory cytokines, presence of infection, prostaglandins and hormones release, matrix metalloproteinase activation^29^. Thus, many factors lead to uterine contractions and premature rupture of fetal and decidua membranes. There are some investigations and reviews about changes of mtCU components expression during skeletal muscle pathology^30^, cancer, cardiovascular^31^ and neurodegenerative disorders^32^. Although it is known that disruption in work of system maintaining calcium homeostasis may provoke PTB^33^, a number of studies concerning mentioned mtCU proteins at PTB is very limited.

Present study allows to explore the phenomenon of mtCU from two sides: from the point of view of gestational dynamics and in terms of PTB pathology in comparison to normal pregnancy.

## Results

### Characteristics of the patients

Сlinical characteristics of all patients (n = 50) involved in study are presented in Table 1. Based on these characteristics women were divided into the following groups: normal pregnancy (CTRL, 37–40 weeks of gestation) and three groups with PTB (22–27, 28–32, 33–36 weeks of gestation).

**Table 1:** Clinical characteristics of patients.

Characteristics	PTB			CTRL
	22–27 weeks	28–32 weeks	33–36 weeks	37–40 weeks
**Number**	11	13	14	12
**Maternal age**, **years**	32.3 ± 4.0	33.3 ± 3.8	31.5 ± 5.1	32.4 ± 4.8
**Gestational age at delivery**, **weeks**	25.8 ± 1.1*	30.4 ± 1.3*	34.5 ± 1.1*	39.0 ± 0.7
**Delivery (Caesarean section/Vaginal)**	8/3	11/2	12/2	12/0

Data is listed as mean ± SD. *p < 0.05 versus control. CTRL - control group (normal pregnancy with 37–40 weeks of gestation), PTB – preterm birth.

### Relative protein level of VDAC1 in tissues

VDAC1 reflects mitochondria content in tissues. We used VDAC1 to actin protein level ratio to assess mitochondria content at placenta and myometrium. Relative expression of VDAC1 was not changed in PTB groups compared to CTRL (Figs S1a,b,g; S2a,b,i).

### Expression of MCU and MCUb in placenta at PTB

**Figure 2:**
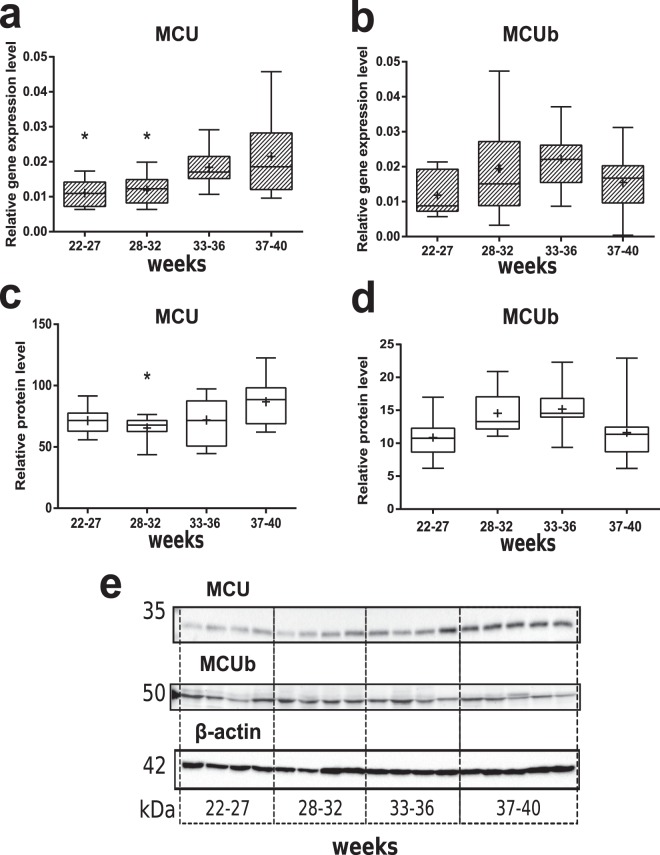
Expression of MCU and MCUb in placenta during gestation. Relative expression level (PCR) of *MCU* (**a**) and *MCUb* (**b**) genes in placenta samples from normal pregnancy (control – 37–40 weeks) and PTB: 22–27 weeks, 28–32 weeks, 33–36 weeks. Relative protein level of MCU (**c**) and MCUb (**d**) (western blot analysis). Representative western blot membranes are shown at panel (**e**) and in Fig. S1. *p < 0.05 versus control. Data is listed as median and interquartile range, mean is shown as a cross (+).

Gene expression of main pore-forming mtCU element - MCU and its paralog - MCUb were evaluated with RT-PCR analysis. We observed that relative mRNA level of *MCU* gene was significantly decreased in placental samples from both 22–27-weeks and 28–33-weeks PTB groups in comparison to CTRL (Fig. 2a). Interestingly, western blot analysis also revealed significant reduction in MCU/β-actin protein level in 28–32-weeks group comparing to normal pregnancies, but not in 22–27-weeks group (Figs 2c,e, S1c). As for MCUb, we did not obtain any changes in its mRNA (Fig. 2b) and protein level normalized to β-actin (Figs 2d,e, S1e) among studied groups. Both MCU and MCUb protein levels normalized to VDAC1 were not changed in PTB as compared to CTRL (Fig. S1d,f).

### Level of MCU and MCUb in myometrium at PTB

**Figure 3:**
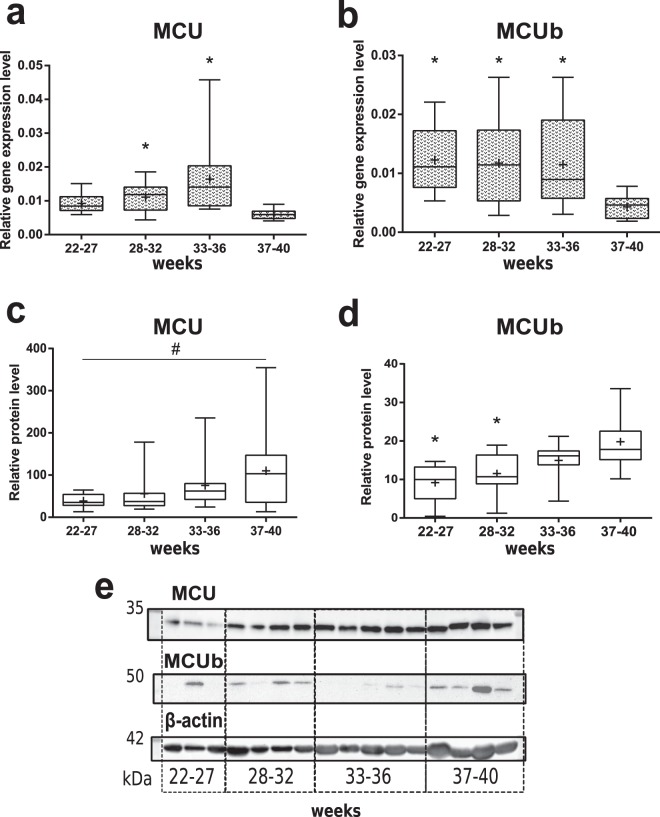
Expression of MCU and MCUb in myometrium during gestation. Relative expression level (PCR) of *MCU* (**a**) and *MCUb* (**b**) genes in myometrium samples from normal pregnancy (control – 37–40 weeks) and PTB: 22–27 weeks, 28–32 weeks, 33–36 weeks. Relative protein level of MCU (**c**) and MCUb (**d**) (western blot analysis). Representative western blot membranes are shown at panel (**e**) and in Fig. S2. *p < 0.05 versus control; ^#^p = 0.07 among studied groups according to Kruskal-Wallis criterion. Data is listed as median and interquartile range, mean is shown as a cross (+).

In contrast to placenta, the opposite changes concerning MCU were observed in myometrium from patients with PTB and women with normal pregnancy. Relative expression level of *MCU* gene was significantly increased in both 28–32-weeks and 33–36-weeks PTB groups in comparison to CTRL (Fig. 3a). Relative MCUb mRNA level was also significantly higher in all PTB groups (22–27-weeks, 28–32-weeks, 33–36-weeks) compared to control group (Fig. 3b). Surprisingly, an upward trend (p = 0.07) of relative MCU/β-actin protein level (Figs 3c,e, S2c,i) was shown among studied groups, although relative MCU/VDAC1 expression was not changed (Fig. S2d). Higher level of MCUb/β-actin in CTRL than in 22–27, 28–32-weeks PTB groups (Figs 3d,e, S2e) was revealed by western-blot analysis, and this pattern persisted at MCUb/VDAC1 normalization (Fig. S2f).

### Level of mtCU regulatory subunits and MICU1/MCU ratio in placenta and myometrium at PTB

In present study we also performed an estimation of *MICU1*, *MICU2* and *SMDT1* relative gene expression, MICU1 protein level and MICU1/MCU ratio in tissue samples from CTRL and PTB groups. MICU2 and SMDT1 protein levels were not analyzed in tissue samples, as verification staining for MICU2 and SMDT1 antibodies did not detected appropriate bands (Fig. S5).

**Figure 4:**
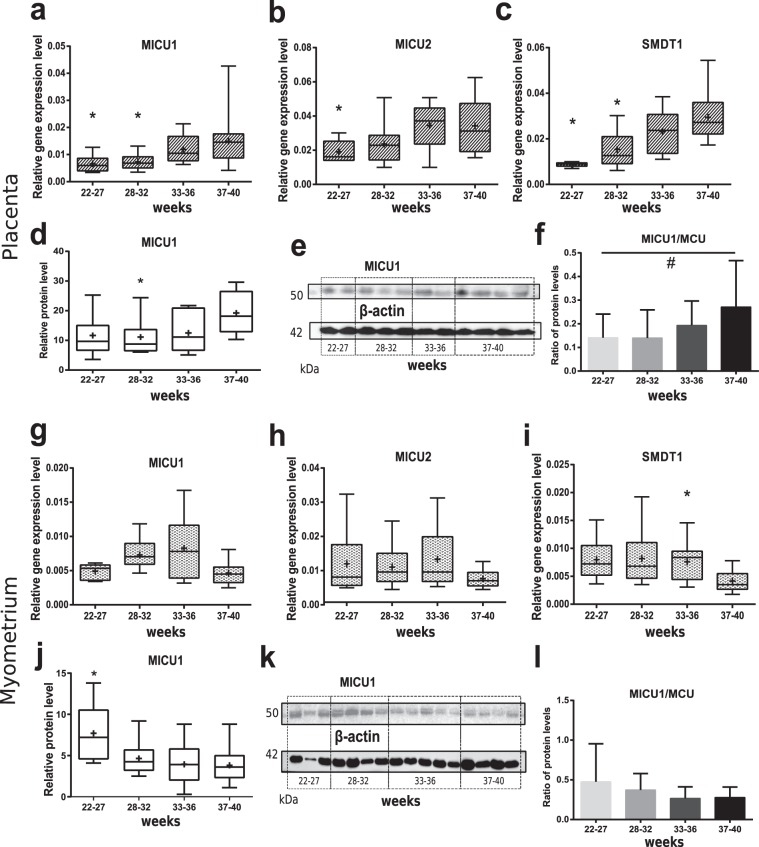
Regulatory subunits level in placenta and myometrium during gestation. Relative expression level (PCR) of *MICU1* (**a** and **g**), *MICU2* (**b** and **h**) and *SMDT1* (**c** and **i**) genes in placenta and myometrium samples from normal pregnancy (control – 37–40 weeks) and PTB: 22–27 weeks, 28–32 weeks, 33–36 weeks. Relative protein level of MICU1 (**d** and **j**) in placental and myometrium samples respectively, representative western blot membranes are shown at panels (e) and (k) and in Figs S1 and S2. MICU1/MCU ratio according to western blot data (**f** and **l**). *p < 0.05 versus control; ^#^p = 0.07 among studied groups according to Kruskal-Wallis criterion. Data is listed as median and interquartile range, mean is shown as a cross (+) (**a**–**d**, **g**–**j**) or as mean ± standard deviation (**f**, **l**).

Similar dynamic as for *MCU* gene was observed among regulatory subunits in placental samples. Expression levels of *MICU1* and *SMDT1* genes, shown in Fig. 4a and c, were significantly decreased in both 22–27 and 28–32 weeks groups compared to 37–40-weeks (normal pregnancy). Relative expression level of *MICU2* subunit gene was significantly down-regulated only in 22–27-weeks group in comparison to control placentas (Fig. 4b). In 33–36-weeks group we did not find the difference in any analysed gene expression level. Western blot analysis displayed lower MICU1 protein expression in 28–32-weeks group than in CTRL in placental tissue both for β-actin (Figs 4d,e, S1h) and VDAC1 (Fig. S1i) normalization.

For myometrium tissue considerable elevation in relative mRNA level was observed for *SMDT1* in 33–36-weeks group compared to 37–40-weeks group (Fig. 4i), whereas no significant differences were found in MICU1 and MICU2 (Fig. 4g,h) gene expression in analysed samples of myometrium. Relative protein content of MICU1 was higher in 22–27-weeks group than in CTRL after β-actin normalization (Figs 4j,k, S2g). MICU1/VDAC1 ratio did not differ among groups (Fig. S2h).

We also calculated the ratio of MICU1 to MCU levels reflecting the rate of subunits cooperation and proposed by Paillard and colleagues^34^. An upward trend (p = 0.07) was shown for this index in placenta (Fig. 4f). For myometrium it did not differ among studied groups (Fig. 4l).

### Calcium content in placenta and myometrium at PTB

**Figure 5:**
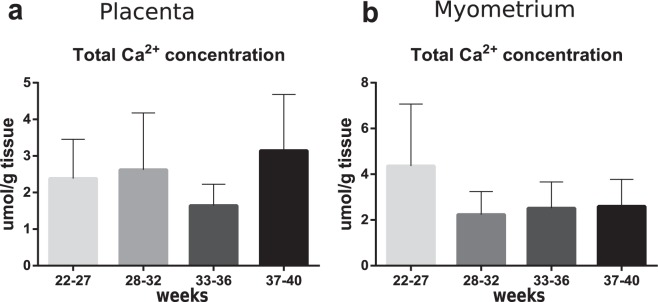
Total Ca^2+^ content in placenta (**a**) and myometrium (**b**) samples from normal pregnancy (control – 37–40 weeks) and PTB: 22–27 weeks, 28–32 weeks, 33–36 weeks. Data is listed as mean ± standard deviation.

Measurement of total calcium content did not reveal any significant differences in this parameter among groups either in placenta or in myometrium tissue (Fig. 5).

### Mitochondrial membrane potential, mtCU subunits protein expression and MICU1/MCU ratio in isolated placental mitochondria

**Figure 6:**
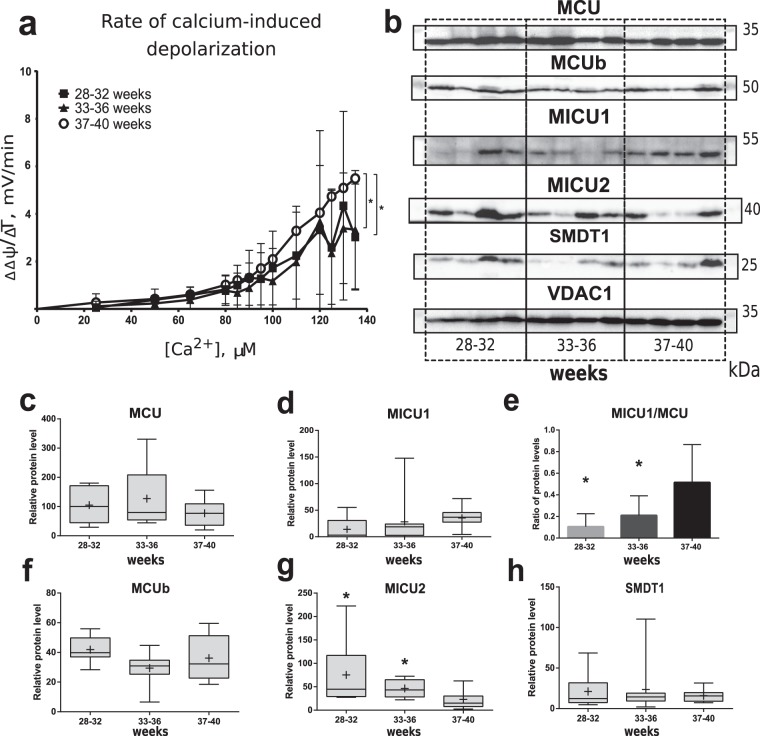
Isolated placental mitochondria membrane potential response to titration with Ca^2+^, mtCU subunits protein level and MICU1/MCU ratio in CTRL (37–40 weeks) and PTB (28–32 weeks, 33–36 weeks) groups. Rate of Ca^2+^-induced depolarization is shown at panel (**a**). Representative western blot membranes are shown at panel (**b**) and in Fig. S4. Relative protein level of MCU (**c**), MICU1 (**d**), MCUb (**f**), MICU2 (**g**) and SMDT1 (**h**). Ratio MICU1/MCU protein level (western blot analysis) (**e**). Full-length blots are presented in Supplementary Figure S6b. *p < 0.05 versus control. Data is listed as mean ± standard deviation (**a**,**e**) or as median and interquartile range, mean is shown as a cross (+) (**c**,**d**,**f**–**h**).

On the next step we estimated the rate of mitochondrial membrane potential changes during titration with CaCl_2_ on isolated mitochondria from placental samples. For this experiment, an appropriate number of samples were set in control and two PTB groups: 28–32 and 33–36 weeks. Although no any differences were found in the basal mitochondrial membrane potential (Fig. S3), we revealed that placental mitochondria isolated from PTB groups had significantly lower rate of depolarization in response to a gradually increasing calcium concentration (Fig. 6a). Parallel western blot analysis concerning mtCU revealed no difference in protein content level of MCU, MCUb, MICU1 and SMDT1 among studied groups (Figs 6b–d,f,h, S4). MICU1/MCU index steadily increases during the course of gestation (Fig. 6e). Relative level of MICU2 protein was up-regulated in PTB groups in comparison to CTRL (Figs 6g, S4).

## Discussion

Role of calcium in the normal course of pregnancy could not be overemphasized. Ca^2+^ is involved in differentiation and aging of trophoblast and its ability to hormone secretion^35,36^. Ca^2+^-induced contraction of the uterus smooth muscle promotes the parturition at normal physiological pregnancy and could be one of the PTB causes in pathology^37^. Calcium transfer across the placenta starts on 12 week of gestation^38^, providing an adequate fetal metabolism and formation of the fetal bones.

Mitochondrial Ca^2+^ import is based on coordinated work of outer, inner and intermembrane mitochondrial membrane proteins and complexes like VDAC1 and mtCU, respectively^39^. In mitochondrial matrix calcium is required for tricarboxylic acid cycle activation and regulation of cytochrome *c* oxidase work^40,41^. That provides the maintaining of adequate respiration chain efficiency and controls cellular oxygen consumption.

In the present study we analyzed the expression level of mtCU subunits in two components of feto-maternal system – placenta and myometrium at full-term delivery and PTB.

Changes of mRNA relative expression and protein level of mtCU subunits in the placenta indicates the changes in mitochondrial calcium uptake through the studied gestation period. Overexpression of only MCU subunit in the cellular model does not reflect a gain of Ca^2+^ uptake^42^, however coordinated increase of MICU1 expression without any changes of MCUb level in placentas from control group could be the sign of elevated mitochondrial Ca^2+^ uptake at 37–40 weeks of gestation^20,43^. This hypothesis is also supported by more slower depolarization rate of mitochondrial membrane of isolated placental mitochondria induced by Ca^2+^ titration at PTB, since previous investigations have documented simultaneous mitochondrial depolarization and calcium uptake^44,45^. At the same time, an important indicator of mtCU cooperation, MICU1/MCU ratio, also increases to the 37–40 weeks of pregnancy in the placental tissue and mitochondria. Paillard and colleagues showed that high MICU1/MCU ratio correlates with MICU1 to MCU association and involved in cooperative intensification of the relative mitochondrial Ca^2+^ uptake^34^. Our results concerning placenta could be interpreted as a sequential increase of mitochondrial calcium transport efficiency during the course of gestation due to increasing fetus requirements in calcium. Such changes could promote more efficient transportation of Ca^2+^ from mother to fetus^46^, and also act as a kind of “buffer”, which allows to avoid sudden changes in transport level^47^. Calcium import depends on transport proteins of syncytiotrophoblast, located between maternal blood and fetus vessels of chorionic villi, and several calciotropic hormones^48^. One of such hormones, vitamin D, is responsible for absorption of calcium and its metabolism in maternal organism and found in two forms: active (1,25(OH)_2_D) and inactive (25(OH)D). Interestingly, that the content of 1-a-hydroxylase, which converts inactive form of provitamin into active, increases throughout pregnancy with its additional expression in placenta^49,50^. Serum level of calcitonin, another calcium-related hormone, is also increased during gestation^51,52^. Alternatively, lower expression and protein level of MICU1 combined with constant SMDT1 protein level at 22–27 and 28–32 weeks as compared to control could also be attributed to mitochondrial dysfunction leading to overload of mitochondria with Ca^2+^, permeability transition pore opening and increased apoptosis in the placenta^43,53^. Excessive SMDT1 is known to perturb the formation of the Ca^2+^-regulated, full mtCU complex^54^. In this context, MICU2 increase in placental mitochondria at PTB could serve as a compensatory mechanism preventing excessive activation of mtCU^55,56^ and mitochondrial calcium overload with consequent apoptosis. If this is the case, mtCU might be considered as a target for the therapy of apoptosis-related placental dysfunction, however careful investigations with cell and animal models should be performed to test this hypothesis.

The main function of the myometrium during labor is the implementation of coordinated contractions for the fetus expulsion from the uterine cavity. We observed a decreased relative gene expression level of MCU, MCUb and SMDT1 subunits in myometrium at full-term delivery. These changes are opposite to the placental samples where relative mRNA levels of MCU and SMDT1 were the highest at 37–40 weeks group and could be the result of different regulation of expression among tissues. In whole, expression of MCU is regulated at transcriptional, post-transcriptional and post-translational levels^57^. The expression of MCU mRNA is positively regulated by the transcription factor CREB, which is highly expressed in non-pregnant myometrium. At 38–39 weeks of gestation it lowers significantly, and the lowest level of CREB is observed during labor^58^. Therefore, the decrease of MCU mRNA and coordinated decrease of MCUb and SMDT in the control group may be the consequence of reduced CREB expression at full-term pregnancy. It is well known that myometrium during pregnancy is subjected to hypertrophic changes. Shynlova and colleagues demonstrated a pronounced hypertrophy of myometrium throughout pregnancy in rat model using caveolin 1 protein, involved in calcium entry and homeostasis, as a marker of myometrial smooth muscle cells^59^. To date, mtCU is considered as a trophic factor for both smooth and skeletal muscles. For instance, MCU down-regulation by overexpression of miR-25 and miR-138 resulted in vascular smooth muscle cells hypertrophy in pulmonary arterial hypertension^60^. On the other hand, increasing content of the MCU protein accompanied by changes in MICU1 and MCUb regulatory subunits during the course of gestation could be a hallmark of intensified regulation of mtCU activity and attributes to the myometrial hypertrophy. A simultaneous decline of the activating MICU1 and an increase of the inhibitory MCUb subunit content could be indicators of decreasing mtCU activity by the full-term gestation. This may be necessary for provision of high concentration of cytoplasmic Ca2 + in myometrial cells and effective uterine contractions during childbirth. Different dynamics of mtCU subunits mRNA and protein content could be also associated with post-translational regulation of these proteins. For instance, MCU is known to be directly phosphorylated and activated with calcium-sensitive proline-rich tyrosine kinase 2 (Pyk2) in rat cardiomyocytes^61^. Although the role of this kinase is not investigated in pregnant and non-pregnant myometrium yet, it was recently shown by Grossi *et al*.^62 that Pyk2^ promotes contractile phenotype in arterial smooth muscle cells. Thus, the exact mechanism of mtCU activity regulation in myometrium during pregnancy remains to be determined.

Changes in mtCU subunits expression could be related to the fluctuations of calcium ions concentration in placenta and myometrium. To verify this idea we performed measurement of total calcium content in samples of studied organs. Since we did not observed changes in total calcium content either in placenta or in myometrium samples between groups, obtained results could be a sign of redistribution of calcium among intracellular compartments rather than oscillations of its content.

Study of the placenta and myometrium in humans at normal pregnancy at different time points seems to be difficult to implement due to ethical reasons. In present work we used PTB group as a way to solve this complexity. We interpret the changes in mtCU components level as events accompanying the second half of pregnancy (from 22 to 40 weeks). An altered level of mtCU subunits at PTB could be otherwise considered as a link between alterations of mitochondrial calcium import and development of pathology.

Normal course of pregnancy and fetal development totally depend on appropriate calcium uptake and the functioning of organelles, involved in its redistribution within the cell, such as endoplasmic reticulum and mitochondria. Further study of the mechanisms of calcium import regulation and calcium sequestration would help in understanding of its role at the different stages of gestation and its involvement in the pathology development.

## Materials and Methods

### Ethics Statement

All procedures and experimental protocols involving myometrium and placental tissue were conducted in accordance with the Declaration of Helsinki, guidelines for Good Clinical Practice and Local Committee on Biomedical Research Ethics of National Medical Research Center for Obstetrics, Gynecology and Perinatology of Ministry of Healthcare of Russian Federation. All experimental protocols were approved by the Commission of Biomedical Ethics at National Medical Research Center for Obstetrics, Gynecology and Perinatology of Ministry of Healthcare of Russian Federation, Moscow (Ethic’s committee approval protocol No13, 6^th^ of December 2013). All the patients signed informed consent in accordance with the Ethics Committee requirements and Helsinki Declaration of the World Medical Association.

### Sample collection

Myometrial and placental samples were collected immediately after vaginal delivery or elective caesarean section proposed on clinical grounds from women with normal pregnancies and women with PTB in Research Center for Obstetrics, Gynecology and Perinatology in Moscow. Myometrial biopsy (0.5 × 0.5 × 0.5 cm) was obtained from the upper edge of lower segment uterine incision and immediately frozen in liquid nitrogen. The central area of placental chorionic tissue was dissected, and the maternal decidua and amniotic membranes were removed. Placental samples were collected from the same area each time (1.5 cm next to the umbilical cord insertion, 1 cm in depth) for reducing the bias caused by differences in gene expression within the same placenta depending on sampling site^63^. Tissue fragments were immediately frozen in liquid nitrogen and stored until use. For mitochondria isolation a piece of placenta was placed in ice-cold physiological saline (0.9% NaCl).

### Isolation of placental mitochondria

Isolation of placental mitochondria was performed by modified method of differential centrifugation described in our previous work^64^. Briefly, blocks of placenta (1.5 × 1.5 cm) were minced at first with scissors and then with blade-type homogenizer in isolation medium (10 mM Tris, 0.25 M sucrose, 0.5 mM EDTA, 0.5 mM EGTA, pH = 7.5), supplemented with 0.1% BSA. Homogenate was centrifuged for 10 min 1,000 g at 4 °C. The supernatant was collected and centrifuged for 17 min 7,200 g at 4 °C to precipitate mitochondria fraction. The pellet was resuspended in isolation medium and suspension was centrifuged under the same conditions. After centrifugation, the supernatant was discarded and the pellet was resuspended in the minimum volume of isolation medium with EGTA. Mitochondrial protein concentration was measured by biuret method^65^.

### Total calcium content measurement

Calcium content in placental and myometrial tissue was measured by measuring absorbance of Ca-arsenazo III complex at 650 nm according to Brown *et al*. with modifications^66^. Calcium-sensitive dye arsenazo III (Sigma Aldrich, US) was diluted to 0.12 mM concentration in buffer containing 400 mM KCl, 1 mM MgCl_2_, 25 mM MOPS, pH = 6.5. 20 μl of tissue homogenate diluted in 5 times in milliQ water was added to 200 μl of arsenazo III solution, incubated for 5 minutes and then the absorbance was measured. Calcium content was calculated using calibration with CaCl_2_ solutions and normalized to the mass of the sample.

### RNA extraction and reverse transcription reaction

Samples of placental and myometrial tissues were homogenized with mortar and pestle in liquid nitrogen. The powder (100–200 mg) was dissolved in 1 ml of Extract RNA Reagent (Evrogen, Russia). All further procedures were carried out according to the manufacturer’s protocol. RNA concentration and 260/280 ratio was measured with spectrophotometer DS-11 (DeNovix, USA). For the reverse transcription reaction, 0.5 μg of total RNA was reverse transcribed using MMLV-RT kits (Evrogen, Russia).

### Real-Time Quantitative RT-PCR

Full experimental procedure is available in Supplementary Material.

### Western blot analysis

Sample preparation and immunoblotting were performed as previously described^64^. Commercial antibodies (Table S1) were tested for entire membrane staining in order to detect the band of interest (Fig. S5). After blotting the gel with loaded samples, Ponceau S was used as reversible stain for detecting protein bands. Membrane was cut with the aim of obtaining a signal of all proteins of the interest from the same run (representative full-size membrane is shown on Fig. S6). After washing in phosphate buffered saline and blocking in 5% milk, membranes were incubated with primary antibodies overnight at 4 °C with gentle shaking. After washing, the membranes were incubated with peroxidase-conjugated secondary antibodies for 1 h at room temperature. Target proteins were detected using Novex ECL Kit (Invitrogen, USA) in ChemiDoc station (Biorad, USA). Optical densities of the protein bands were measured using ImageLab Software. Relative protein expression level was determined as the ratio of optical densities of target protein to the internal reference protein: β-actin and VDAC1 for placenta and myometrium tissues and VDAC1 for isolated placental mitochondria. For placenta and myometrium data normalized to β-actin is presented in the text, and data normalized to VDAC1 is presented in Supplementary Material. Labeling of western blot images were conducted in Inkscape software with minimal processing. Samples from all patients involved in the study were analysed on three separate blots at least.

### Determination of placental mitochondria sensitivity to Ca^2+^ exposure

Mitochondrial ∆Ψ measurement was performed in mitochondrial suspension through fluorescence changes of lipophilic cationic dye safranin O at 495/586 nm excitation/emission wavelengths, recorded with Cary Eclipse fluorescence spectrophotometer (Agilent Technologies, USA). Additional information is provided in Supplementary Material.

### Statistical analysis

Data is presented as mean ± standard deviation (SD) and median with interquartile range. The Shapiro-Wilk normality test was used to estimate distribution. One-way analysis of variance (ANOVA) followed by the Tukey’s post-hoc test was used to identify differences among multiple groups with normal distribution. One-way Kruskal-Wallis non-parametric ANOVA followed by the post-hoc Dunn test was used to calculate statistical differences for non-normal distributions. All calculations were performed by Prism 7.0 software (GraphPad, USA) and Website VassarStats for Statistical Computation (www.vassarstats.net). P-value < 0.05 was considered significant and was indicative of the differences in comparison to control.

## Supplementary information

Supplementary material
