# Supplementary material for: Gestation age-associated dynamics of mitochondrial calcium uniporter subunits expression in feto-maternal complex at term and preterm delivery
Source: Sci Rep. 2019 Apr 2;9:5501. doi: 10.1038/s41598-019-41996-3 (PMC6445111; doi:10.1038/s41598-019-41996-3)
Supplement: Supplementary file 1 — Supplementary material [file 41598_2019_41996_MOESM1_ESM.docx]

**SUPPLEMENTARY MATERIAL**

**Gestation age-associated dynamics of mitochondrial calcium uniporter subunits expression in feto-maternal complex at term and preterm delivery**

Polina A. Vishnyakova, Nadezhda V. Tarasova, Maria A. Volodina, Daria V. Tsvirkun, Iuliia A. Sukhanova, Tatiana A. Kurchakova, Nataliya E. Kan, Marzanat K. Medzidova, Gennadiy T. Sukhikh and Mikhail Yu. Vysokikh

Corresponding author: Polina A. Vishnyakova,

Laboratory of mitochondrial medicine, Research Center for Obstetrics, Gynecology and Perinatology, Ministry of Healthcare of the Russian Federation, 4, Oparina street, Moscow, 17513, Russia. Tel: +79150658577. Email: [vpa2002@mail.ru](mailto:vpa2002@mail.ru) or [vishnyakovapolina@gmail.com](mailto:vishnyakovapolina@gmail.com)

**SUPPLEMENTAL RESULTS**


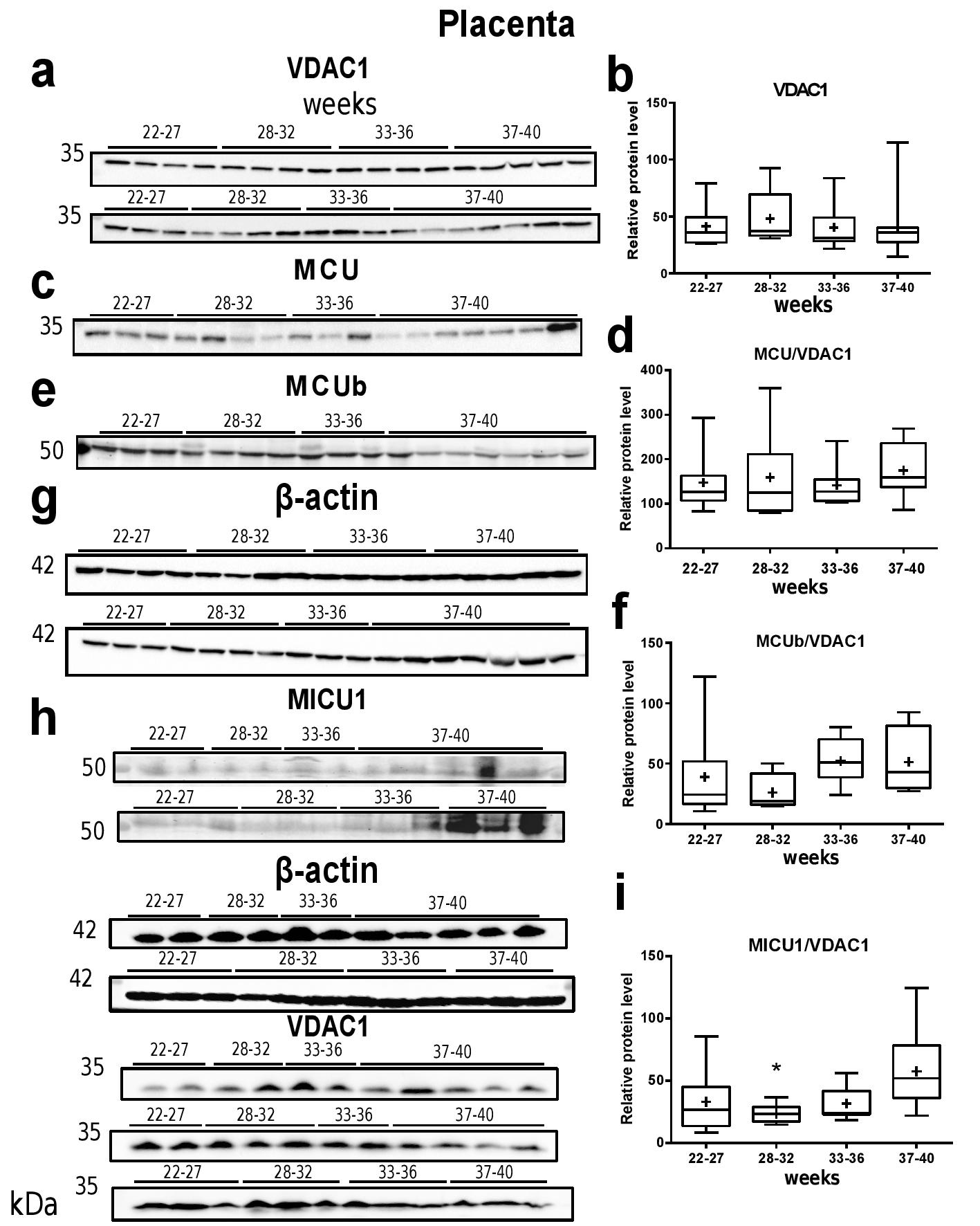


Fig.S1. Representative western blot membranes (a, c, e, g, h) and relative expression level of studied proteins (b, d, f, i) from all the rest placental samples are shown. Data is listed as median and interquartile range, mean is shown as a cross (+). * – p < 0.05 versus control (37-40 weeks).


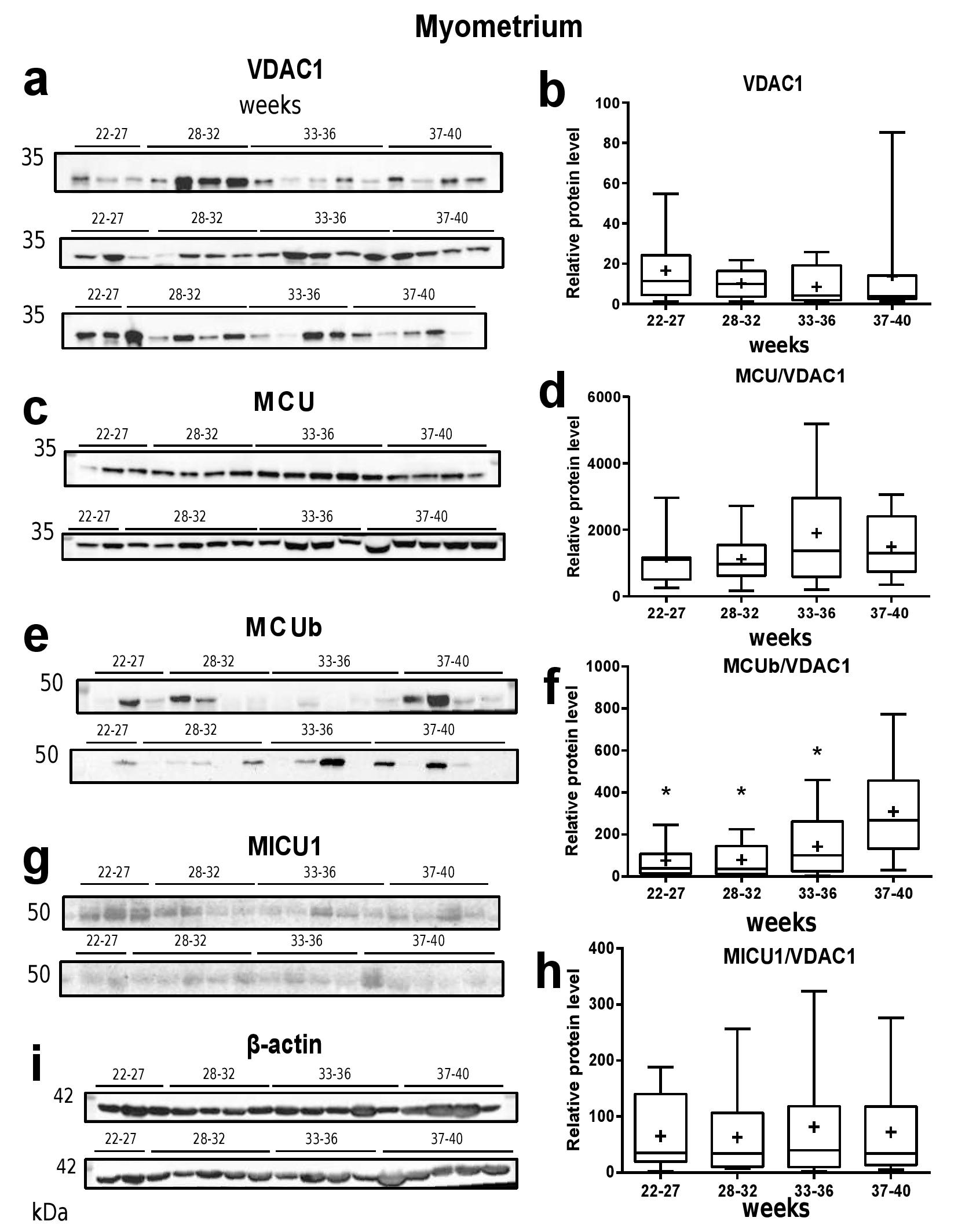


Fig.S2. Representative western blot membranes (a, c, e, g, i) and relative expression level of studied proteins (b, d, f, h) from all the rest myometrium samples are shown. * – p < 0.05 versus control (37-40 weeks).

**
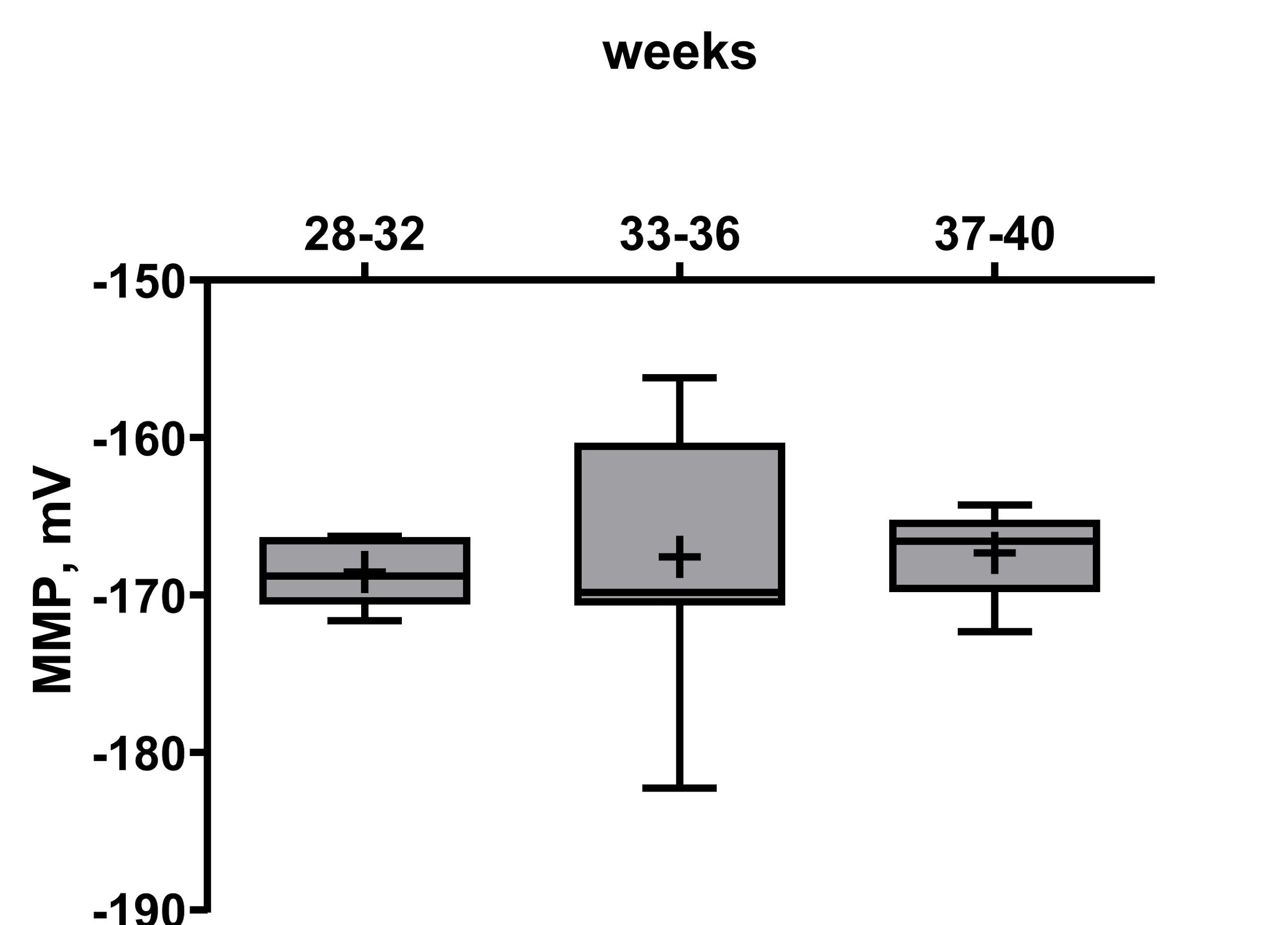
**

Fig.S3. Calculated baseline mitochondrial membrane potential (MMP) of isolated placental mitochondria.


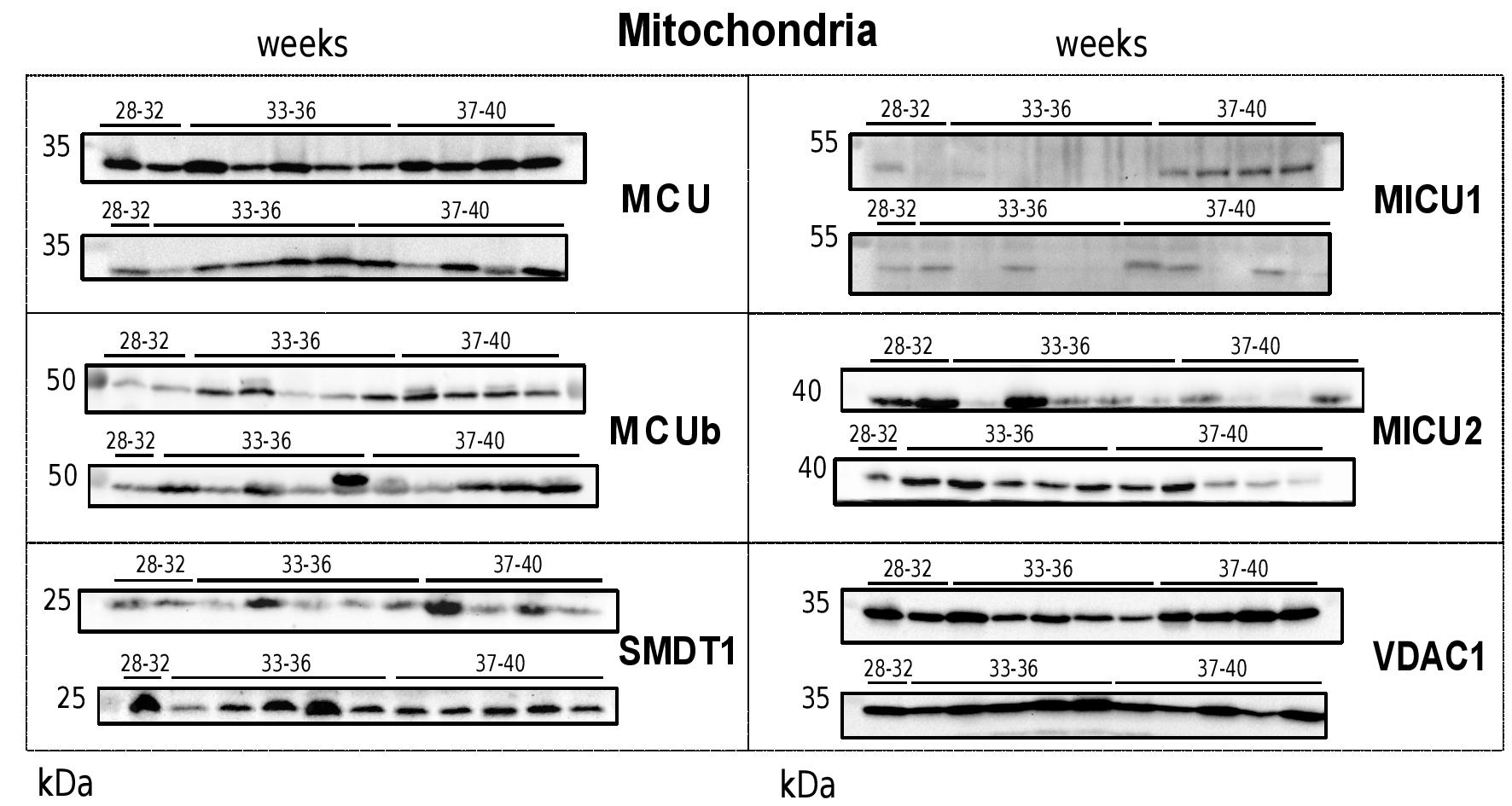


Fig.S4. Representative western blot membranes from all the rest placental mitochondrial samples are shown.

**SUPPLEMENTAL METHODS**

**Real-Time Quantitative RT-PCR.** Quantification of mRNA was performed using a DT-96 thermocycler (DNA-Technology LLC, Russia). Real-time PCR reactions were conducted in a reaction volume of 10 μl, containing 50 ng of cDNA, 300 nM of each primer and 2 μl of 5xSybrGreen-mix (Evrogen, Russia) in triplicate. All primer sequences were generated and verified for specificity by Primer-BLAST: *ACTB:* F – GAGCGGGAAATCGTGCGTGACATT, R – GATGGAGTTGAAGGTAGTTTCGTG (Product size – 234 bp); *MCU:* F – ATTGCTCAGGCAGAAATGGAC, R – AGGCAAACAGGTGTTCCTTCT (Product size – 134 bp); *MICU1:* F – ACTGTGATGGCAATGGCGAA, R – TAAAGCGAAGTCCCAGGCAG (Product size – 158 bp)**;** *MICU2:* F – AGAGACCTTGGCGATAAAGGG, R – ATCCAGAATGGGGTTTAGTGAGG (Product size – 82 bp); *SMDT1:* F – CGGGCCTAGCTTGAGGAAAG, R – AGGCCGAAGGACATTTTCACC (Product size – 138 bp); *MCUB:* F – CTCCGCCCCAGGTTTTGC, R – AGGTGGCACCACGGTACTAT (Product size – 83 bp). Agarose gel (1.5%) electrophoresis and melting curve analysis were used for amplicon size estimation and primer specificity. The PCR program consisted of an initial step at 95°C for 5 min, followed by 45 cycles of denaturation at 95°C for 10 s, annealing at 60°C for 20 s and elongation at 67°C for 20 s, followed by melting at a gradient from 65°C to 95°C. Relative gene expression was determined as the ratio of the target gene to the internal reference gene expression (β -actin) based on Ct values using QGENE software.

Table S1. Antibodies used in Western blot analysis.

| Antigen | Catalog number | Manufacturer | Description | Dilution |
| --- | --- | --- | --- | --- |
| MCU | ab121499 | Abcam, USA | Rabbit PC | 1/1000 |
| MICU1 | ab102830 | Abcam, USA | Rabbit PC | 1/250 |
| MICU2 | STJ114090 | St John's Laboratory, | Rabbit PC | 1/2000 |
| MCUb | 20387-1-AP | Proteintech, USA | Rabbit PC | 1/700 |
| SMDT1 | orb156017 | Biorbyt, UK | Rabbit PC | 1/500 |
| VDAC1 | ab154856 | Abcam, USA | Rabbit MC | 1/2000 |
| Beta-actin | A5441 | Sigma, USA | Mouse MC | 1/5000 |


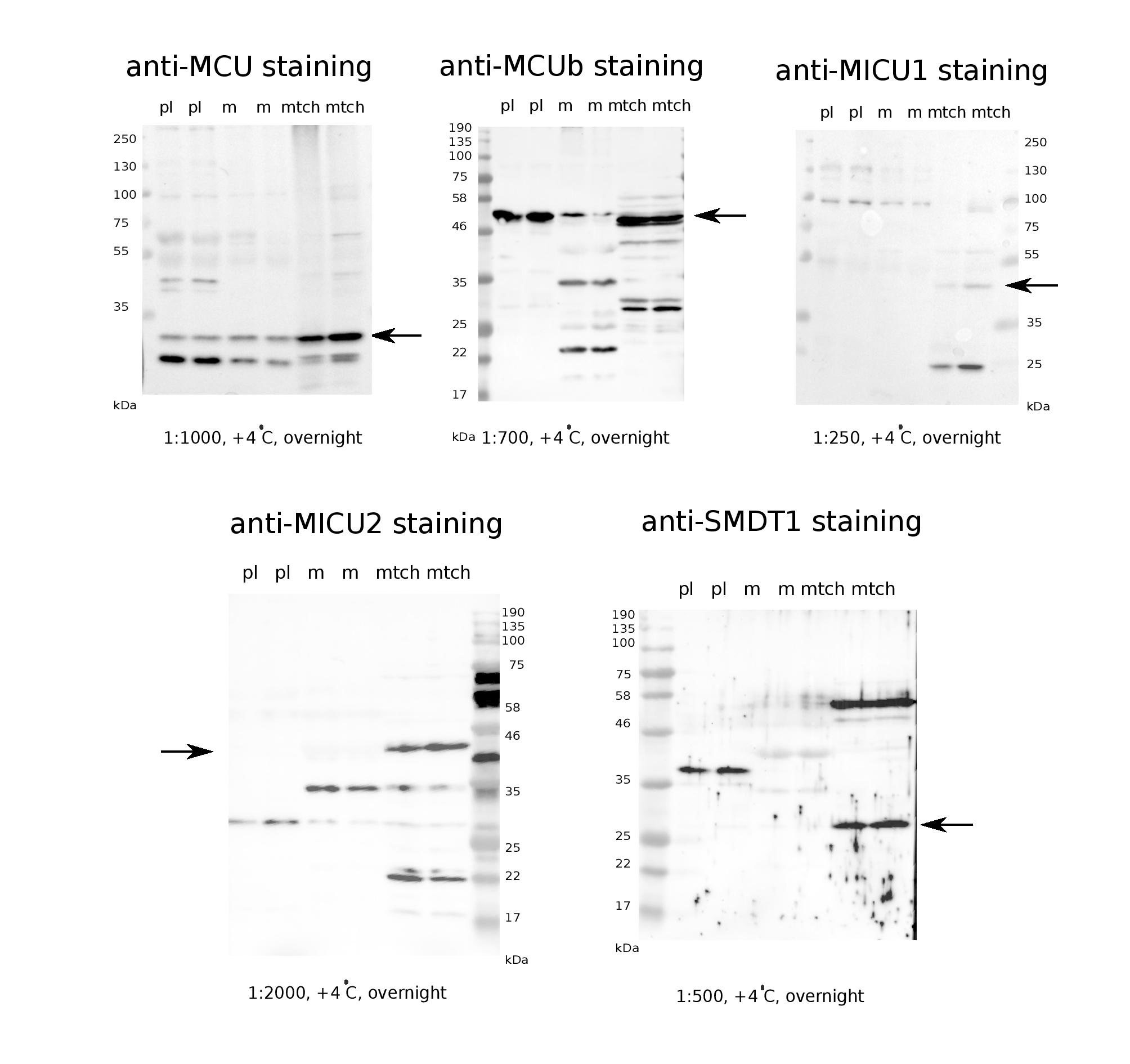


Fig.S5. Verification of anti-MCU, anti-MCUb, anti-MICU1, anti-MICU2, anti-SMDT1 antibodies. Random samples of placenta (pl), myometrium (m) and placental mitochondria (mtch) were used. Incubation conditions are indicated. Arrows point the band of interest.


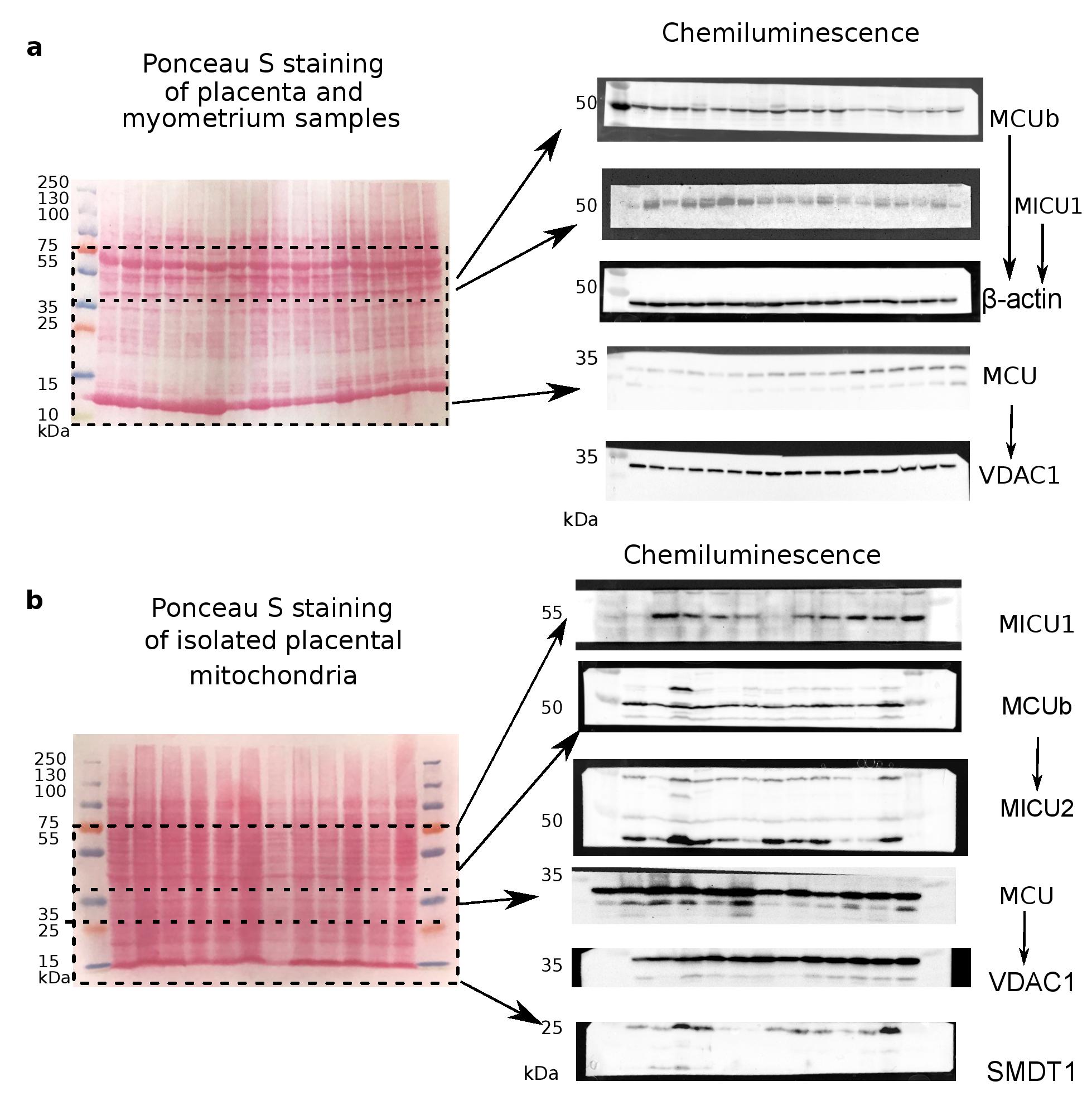


Fig.S6. Full-size membrane after blotting of polyacrylamide gel. Schemes of membrane Ponceau S staining and cutting of placenta/myometrium samples (a) and isolated placental mitochondria (b) samples are shown. After visualization of the proteins with Ponceau S membranes were cut as indicated with dotted line. Samples from all studied groups were loaded on the same gel.

**Determination of placental mitochondria sensitivity to Ca^2+^ exposure.** Mitochondrial ∆ᴪ measurement was performed in mitochondrial suspension through fluorescence changes of lipophilic cationic dye safranin O at 495/586 nm excitation/emission wavelengths, recorded with Cary Eclipse fluorescence spectrophotometer (Agilent Technologies, USA). Detailed protocol is described in our previous work (Vishnyakova et al., 2016). Fluorescent data were quantified using titration of mitochondrial suspension with K+ in the presence of valinomycin with further fitting of the calibration curve for safranin signal to second-order polynomial equation, as was suggested by Figueira et al (Figueira et al., 2012). Representative recording of K+ titration and fitted calibration curve are presented in Fig.S7 a, b.


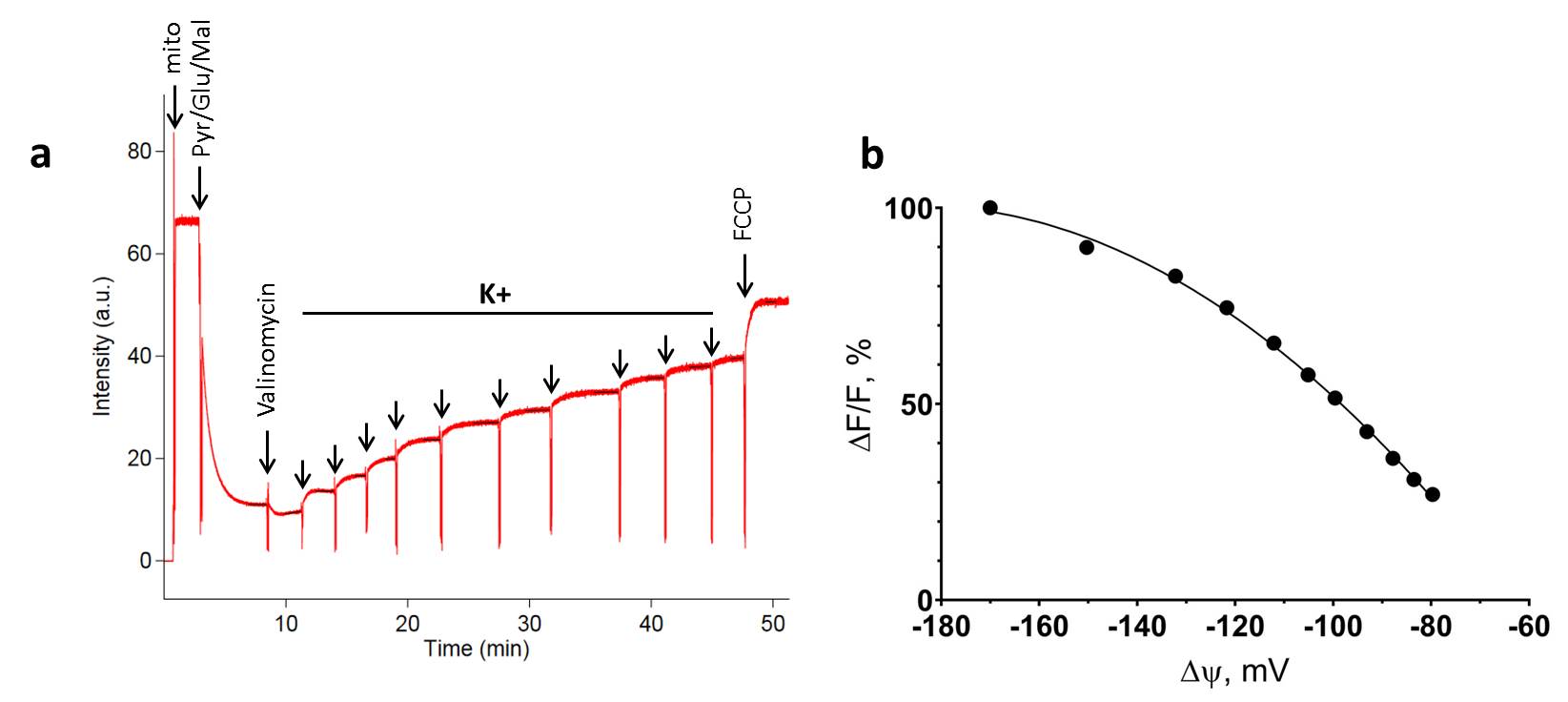


Fig.S7. Representative recording of titration of isolated placental mitochondria suspension with K+ (a) and (b) corresponding calibration curve (dots represent actual 𝚫F/F values, line - 2nd order polynomial fitting equation).

**References**

Figueira, T. R., Melo, D. R., Vercesi, A. E., and Castilho, R. F. (2012). “Safranine as a Fluorescent Probe for the Evaluation of Mitochondrial Membrane Potential in Isolated Organelles and Permeabilized Cells,” in (Humana Press), 103–117. doi:10.1007/978-1-61779-382-0_7.

Vishnyakova, P. A., Volodina, M. A., Tarasova, N. V., Marey, M. V., Tsvirkun, D. V., Vavina, O. V., et al. (2016). Mitochondrial role in adaptive response to stress conditions in preeclampsia. *Sci. Rep.* 6, 32410. doi:10.1038/srep32410.
